# The association between the experience of lay responders and response interval to medical emergencies in a rural area: an observational study

**DOI:** 10.1186/s12873-023-00803-z

**Published:** 2023-05-06

**Authors:** S. M. Starck, J. J. Jensen, L. Sarkisian, H. Schakow, C. Andersen, F. L. Henriksen

**Affiliations:** 1 Research Unit of Cardiology, Department of Cardiology, Odense University Hospital, J.B. Winsløws Vej 4, 5000 Odense C, Denmark; 2 Emergency Medical Services, Region of Southern Denmark, Damhaven 12, 7100 Vejle, Denmark; 3 Department of Anaesthesiology, Odense University Hospital, J.B. Winsløws Vej 4, 5000 Odense C, Denmark

**Keywords:** Response interval, Response time, Community first responder, Automated external defibrillator, AED, Bystander defibrillation

## Abstract

**Aim:**

The aim of this retrospective observational study was to determine how response intervals correlated to the experience of the community first responders (CFRs) using data collected from the Danish Island of Langeland via a global positioning system (GPS)-based system.

**Methods:**

All medical emergency calls involving CFRs in the time period from 21st of April 2012 to 31st of December 2017 were included. Each emergency call activated 3 CFRs. Response intervals were calculated using the time from when the system alerted the CFRs to CFR time of arrival at the emergency site measured by GPS. CFRs response intervals were grouped depending on their level of experience according to ≤ 10, 11–24, 25–49, 50–99, ≥ 100 calls accepted and arrived on-site.

**Results:**

A total of 7273 CFR activations were included. Median response interval for the CFR arriving first on-site (*n* = 3004) was 4:05 min (IQR 2:42–6:01) and median response interval for the arrival of the CFR with an automated external defibrillator (*n* = 2594) was 5:46 min (IQR 3:59–8:05). Median response intervals were 5:53 min (3:43–8:29) for ≤ 10 calls (*n* = 1657), 5:39 min (3:49–8:01) for 11–24 calls (*n* = 1396), 5:45 min (3:49–8:00) for 25–49 calls (*n* = 1586), 5:07 min (3:38–7:26) for 50–99 calls (*n* = 1548) and 4:46 min (3:14–7:32) for ≥ 100 calls (*n* = 1086) (*p* < 0.001). There was a significant negative correlation between experience and response intervals (*p* < 0.001, Spearman’s rho = -0.0914).

**Conclusion:**

This study found an inverse correlation between CFR experience and response intervals, which could lead to increased survival after a time-critical incident.

## Introduction

Critically illness should be treated as fast as possible in case of life-threatening events such as sudden cardiac arrest to improve their odds for better mental outcome and survival. To shorten response intervals in areas with prolonged arrival times for emergency medical services (EMS), it has been proposed that citizens trained as community first responder (CFRs) can be activated to perform cardiopulmonary resuscitation (CPR) before EMS arrival. A randomized study carried out in Sweden showed an absolute increase of 14% in bystander-initiated CPR with the use of a global positioning system (GPS)-based message system [1]. Other lay responder systems have been successfully used in the Netherlands [2, 3], Switzerland [4], France [5] and are under development in the United Kingdom [6] and North America [7].

The global resuscitation alliance postulated a *frame of survival* as an extension of the chain of survival. The frame of survival states that leadership and training are needed to improve the quality of resuscitation and thereby lead to a culture of excellence [8]. One way to improve quality is faster initiation of CPR and we theorize that more experienced and trained CFRs arrive faster at the emergency site. We assume that more experienced CFRs would have increased knowledge of the location of the local AED-cabinets and that they would have established routines to arrive quicker at the site. On the Danish island of Langeland, a GPS-based community first responder system was initiated in 2012 to locate and dispatch CFRs alongside standard emergency medical services response [9].

In this retrospective observational study, our main purpose was to determine a possible correlation between response intervals and experience of the community first responders by using data collected from the island of Langeland.

## Methods

### Study design and approval

This retrospective observational study presents data collected from a GPS-based system on the Danish Island of Langeland. Approval for this study was given by The Danish Data Protection Agency (Journal no. 17/32047) and Danish Patient Safety Authority under the administration of Danish Health Authority (no. 3–3013-2848/1, ref.LOSC) and the volunteers accepted during their registration in the project, that data was collected from their smartphones, stored on the server and used for research purposes. In Denmark, ethical approval is not needed for this kind of study.

### Setting

The island of Langeland has a population of approximately 12,000 inhabitants, but during summertime approximately 260,000 tourists visit the holiday island. It is 52 km long and 11 km at its widest and is bridge connected to the mainland [9]. There are one town and four larger villages, and the population is a typical island community, where most of the island’s inhabitants have lived most of their life on the island. Langeland has one ambulance and one paramedic vehicle and in case the ambulance is reserved for another duty, response intervals increase drastically as another ambulance needs to arrive from the city of Svendborg placed on the mainland 20–50 km away [9]. The island has 96 AEDs placed strategically with no more than 2 km between each of them.

### GPS system

A GPS-based system to activate CFRs was initiated in 2012 on Langeland. Each time an ambulance was dispatched to an emergency (not only cardiac arrest), the system identified the nine closest CFRs in a five km radius from the emergency site via GPS and notified them by an alarm sent to their smartphone. The CFRs could then choose to accept or decline the call. Based on the choices made, the system within 20 s identified the three closest CFRs and sent a map to the location of the emergency site and tasks. Two CFRs were dispatched directly to the emergency site, while the third was sent to pick up an automated external defibrillator (AED) before proceeding to the emergency site. These tasks are selected by the system based on the CFRs GPS location and nearest AED. After each activation, there was a possibility for a debriefing for the CFRs [9, 10].

### Recruitment of lay responders

CFRs were recruited to the project through local advertisement and were mostly laypersons but included some off-duty healthcare providers. To become CFR certified, the CFRs needed to be at least 18 years of age and complete a 12-hour basic life support training course provided by Langeland AED Association. The CFRs were trained by ERC certified staff. Each CFR is manually approved by an administrator before being able to accept assignments in the system. To remain active as a CFR an annual 3-hour retraining course is required.

### Data collection

All medical emergency calls involving CFRs on Langeland in the time period from 21st of April 2012 to 31st of December 2017 were included. CFRs were excluded from analysis if they did not arrive on-site or if their data did not appear to be appropriate (arrived on-site > 2 hours after accepting the call).

The time for arrival at the emergency site was automatically logged by the GPS-based system. Time and date for when the system alerts the CFRs, the emergency site’s address and CFRs arrival time at the emergency site were collected from the project-server. Emergency service records were acquired and were used to correct any missing data as far as possible in the CFR information.

### Definition of variables

CFR experience was measured as the number of times a CFR was activated and arrived to the emergency site, and was divided into the following groups based on milestones from the Langeland AED Association as the association gives rewards based on the categories: ≤ 10, 11–24, 25–49, 50–99, ≥ 100 calls accepted. We presumed these milestones to be applicable and good indicators for CFR experience. These groups were used to investigate the link between CFRs experience and response interval; e.g. the response intervals of a CFR who was called out 34 times was placed in the category ≤ 10 calls the first 10 times he/she was called out, then placed in the category 11–24 calls for the next 14 calls, and finally placed in the category 25–49 for the last 10 calls. Response intervals were calculated using the time from alerting the CFRs to CFRs time of arrival at the emergency site. In the multiple regression analysis, the analysis was carried out without the described experience groups and instead data were re-assessed with the number of each CFR's responses analyzed as a continuous variable instead of in groups.

### Variables of interest

The median response interval could be influenced by multiple variables and was therefore analysed by the effect of a CFR acquiring an AED on the way to the emergency site, the time of day, season and the town of Rudkoebing. Time of day was categorised according to hours at work (7:00 to 15:00), spare time (15:00 to 23:00) and nighttime (23:00 to 7:00). The season was defined meteorologically as winter (December, January, February), spring (March, April, May), summer (June, July, August) and fall (September, October, November). Rudkoebing is the biggest town on Langeland, and therefore potentially had shorter distances to emergencies which may have had an impact on response intervals when compared to the rest of Langeland.

### Statistics

The data were analysed using Stata 16 and statistical significance was set to *p* = 0.05. To validate if data were normally distributed a Q-Q plot was used. Non-normally distributed outcomes were reported as median with interquartile ranges (IQR). Categorical data were described as absolute numbers and percentages. A Spearman’s rank-order correlation was used to assess if there was a monotonic relationship between experience and response intervals. To evaluate non-normally distributed outcomes, a Kruskal Wallis test was used if there were three or more unmatched groups and a Mann–Whitney U-test was used to test for statistically significant differences between two groups. A multiple regression analysis was used to analyse the association between CFR response intervals and the independent variables (experience, AED, year, time of day, season, Rudkoebing vs. the rest of Langeland). A post-hoc analysis was used to determine if the correlation between experience and VFR response interval still prevailed when using only data from the rural region of Langeland and with only data from the town of Rudkoebing.

## Results

**Fig. 1:**
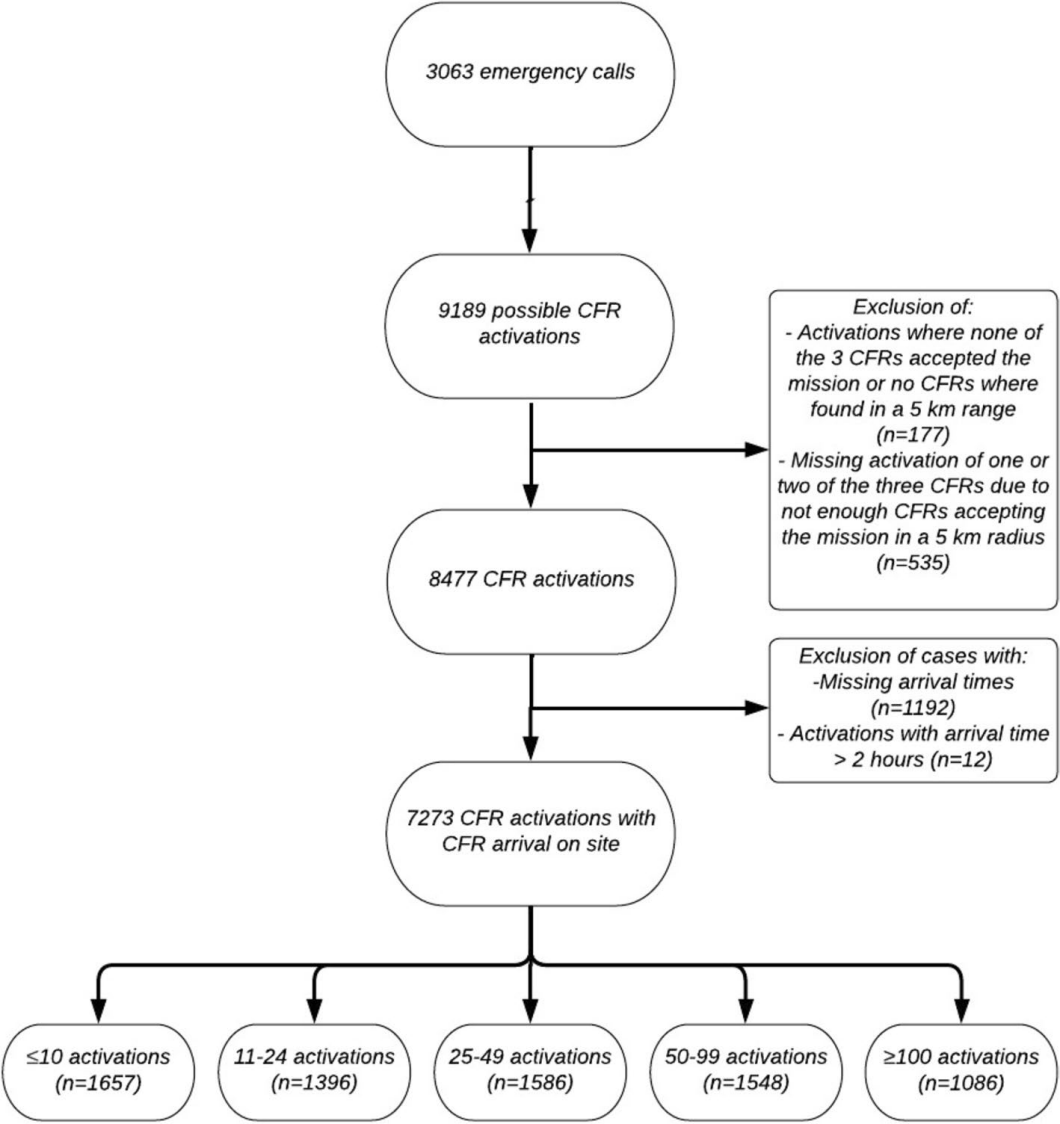
Flow-chart showing the inclusion–exclusion process for CFRs experience

Our study included 3063 emergency calls with activated CFRs resulting in 9189 possible CFR activations, of which 7273 activations were included and 1916 were excluded. We excluded 712 activations with no response intervals generated, 177 activations had no response intervals available because no willing CFRs were available within a five kilometre search radius. The remaining 535 possible activations had no response intervals due to only one or two of the three potential CFRs being available within a five kilometre radius. The CFRs did not arrive on-site in 1192 activations and were thus excluded. We also excluded 12 activations with a response interval over 2 hours (Fig. 1).

At least one CFR arrived at the emergency site in 3004 emergency calls (98.1%). An AED arrived at the emergency site in 2594 emergency calls (84.7%). The annual mean number of alerts for each CFR in the study period was approximately 6.25 alerts divided between 204 CFRs.

**Fig. 2:**
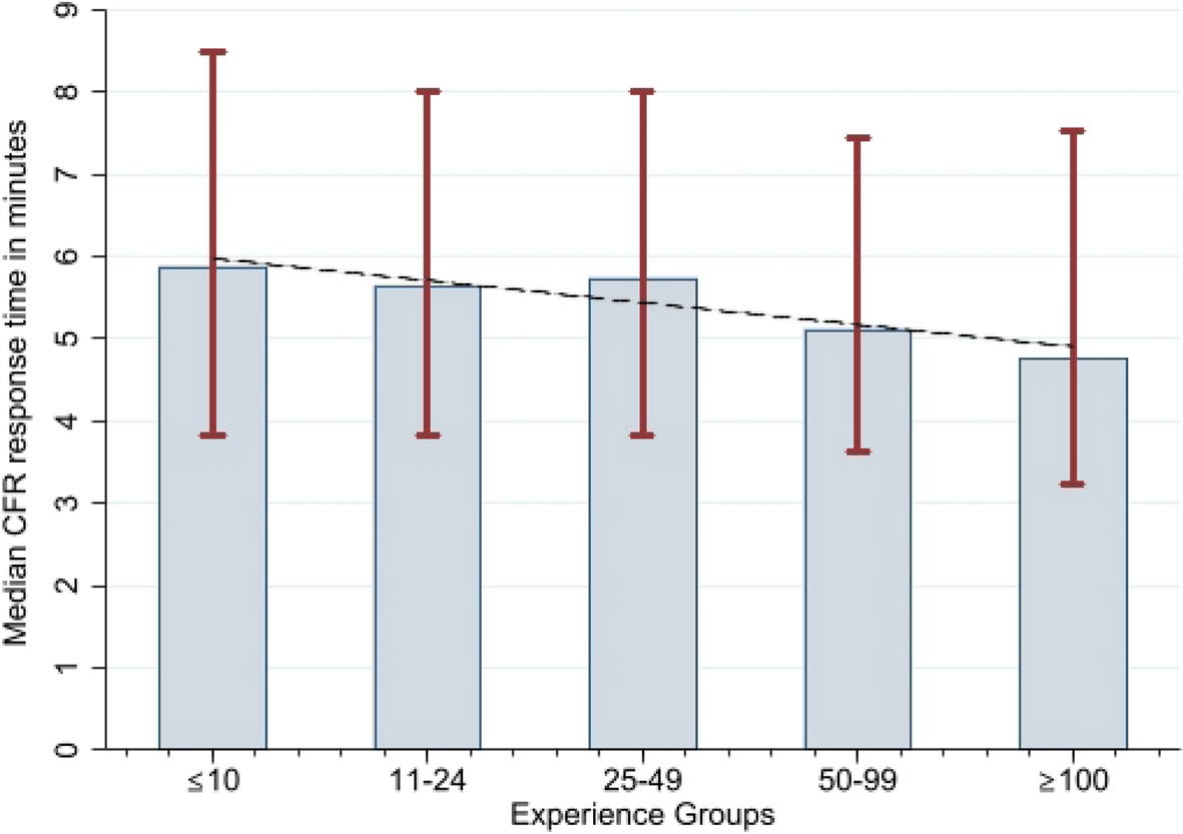
Median community first responder response interval in seconds for each of the five experience groups. The red spikes indicate the 25^th^ interquartile to the 75^th^ interquartile. The black line is a linear prediction plot for the median community first responder response interval. *p* < 0.001

The median response interval for the CFRs who arrived first on-site (*n* = 3004) was 4:05 min (IQR 2:42–6:01). Median response interval for the arrival of the CFRs with an AED (*n* = 2594) was 5:46 min (IQR 3:59–8:05). The median response intervals for all CFRs (*n* = 7273) was 5:29 min (IQR 3:41–7:54). CFRs response intervals generally decreased with increasing experience when divided into the five experience groups (*p* < 0.001) (Table 1; Fig. 2). Likewise, the median response intervals generally decreased with experience when analysed according to subgroups: picking up an AED and time of day. Median response intervals for all CFRs were lowest if the CFR did not run for an AED (5:18 vs 5:46), during spare time (5:03 vs 5:13 at work hours and 7:07 at nighttime) (Table 2). CFR response intervals according to season were 5:26 min in spring, 5:40 min during summer, 5:28 min in fall and 5:23 min winter.

**Table 1:** Summary of important outcomes. Median response intervals are represented in minutes:seconds with interquartile ranges

Outcome	n (%)	Median time in minutes:seconds (IQR)
First CFR on-site	3005 calls (98.1)	4:05 (2:42–6:01)
CFR with AED on-site	2594 calls (84.7)	5:46 (3:59–8:05)
Median CFR response interval	7273 activations (100)	5:29 (3:41–7:54)
Experience groups^*^		
· ≤ 10	1657 activations (22.8)	5:53 (3:43–8:29)
· 11–24	1396 activations (19.2)	5:39 (3:49–8:01)
· 25–49	1586 activations (21.8)	5:45 (3:49–8:00)
· 50–99	1548 activations (21.3)	5:07 (3:38–7:26)
· ≥ 100	1086 activations (14.9)	4:46 (3:14–7:32)

^*^An asterisk represents significant results with a *p*-value of less than 0.0001

**Table 2:** Summary of median response intervals in each experience group for the variables of interest. Median response intervals are represented in minutes:seconds with interquartile ranges

CFR activations	0–10	11–24	25–49	50–99	100 +	All activations	*p*-value
All CFR	5:53 (IQR 3:49–8:29) *n* = 1657	5:39 (IQR 3:49–8:01) *n* = 1396	5:45 (IQR 3:49–8:00) *n* = 1586	5:07 (IQR 3:38–7:26) *n* = 1548	4:46 (IQR 3:14–7:32) *n* = 1086	5:29 (IQR 3:41–7:54) *n* = 7273	0.0001
CFR with an AED	6:12 (IQR 4:25–8:44) *n* = 505	6:00 (IQR 4:17–8:30) *n* = 458	5:59 (IQR 4:11–8:03) *n* = 521	5:23 (IQR 3:52–7:35) *n* = 582	5:01 (IQR 3:21–7:43.5) *n* = 524	5:46 (IQR 3:59–8:05) *n* = 2590	0.0001
CFR without an AED	5:42 (IQR 3:34.5–8:22) *n* = 1152	5:22 (IQR 3:37–7:47) *n* = 938	5:32 (IQR 3:35–7:55) *n* = 1065	5:02 (IQR 3:26–7:18) *n* = 966	4:35 (IQR 3:04–7:25) *n* = 562	5:18 (IQR 3:30–7:45) *n* = 4683	0.0001
Work hours (7:00 to 15:00)	5:37 (IQR 3:34–8:15) *n* = 686	5:19 (IQR 3:28–7:38) *n* = 617	5:44 (IQR 3:54–7:41) *n* = 643	4:51 (IQR 3:18–7:06) *n* = 635	4:28 (IQR 3:02–6:57) *n* = 469	5:13 (IQR 3:27–7:31) *n* = 3050	0.0001
Spare time (15:00 to 23:00)	5:27 (IQR 3:41–7:34) *n* = 686	5:12 (IQR 3:39–7:16) *n* = 530	5:01 (IQR 3:23–7:04) *n* = 624	4:40 (IQR 3:24–7:00) *n* = 559	4:40 (IQR 3:02–6:58) *n* = 371	5:03 (IQR 3:27–7:13) *n* = 2770	0.0002
Nighttime (23:00 to 7:00)	7:45 (IQR 5:40–10:06) *n* = 285	7:40 (IQR 5:26–10:19) *n* = 249	7:11 (IQR 5:03–9:42) *n* = 319	6:31 (IQR 4:39–8:51) *n* = 354	6:02 (IQR 3:56–9:15) *n* = 246	7:07 (IQR 4:52–9:35) *n* = 1453	0.0001
Spring	5:58 (IQR 3:49–8:20) *n* = 393	5:06 (IQR 3:25–7:02) *n* = 339	5:35 (IQR 3:41–7:40) *n* = 455	5:09 (IQR 3:48–7:33) *n* = 341	4:58 (IQR 3:24–7:42) *n* = 234	5:26 (IQR 3:40–7:44) *n* = 1762	0.0038
Summer	5:56 (IQR 3:54–8:44) *n* = 498	6:05 (IQR 4:02–8:36) *n* = 357	5:46 (IQR 3:55–8:04) *n* = 396	5:07 (IQR 3:30–7:40) *n* = 481	5:12 (IQR 3:31–8:23) *n* = 270	5:40 (IQR 3:47–8:12) *n* = 2002	0.0131
Fall	5:44 (IQR 3:49–8:25) *n* = 447	5:39 (IQR 4:03–8:26) *n* = 370	5:49 (IQR 3:43–8:12) *n* = 386	5:09 (IQR 3:42–7:28) *n* = 368	4:23 (IQR 3:01–7:11) *n* = 315	5:28 (IQR 3:42–7:59) *n* = 1886	0.0001
Winter	5:53 (IQR 3:41–8:20) *n* = 319	5:47 (IQR 3:51–7:53) *n* = 330	5:45 (IQR 3:57–8:11) *n* = 349	5:05 (IQR 3:31–6:47) *n* = 358	4:23 (IQR 3:04–6:54) *n* = 267	5:23 (IQR 3:36–7:38) *n* = 1623	0.0001

**Fig. 3:**
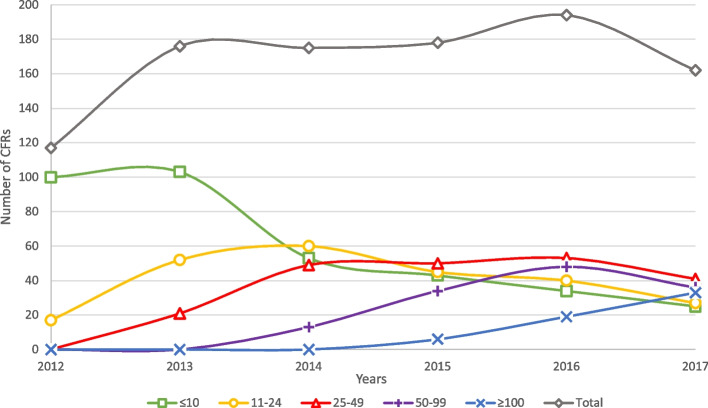
The annual number of VFRs in each experience group. The grey line represents the total annual number of active CFRs on Langeland. The green line represents the group with 10 or less activations, the yellow line represents the group with 11 to 24 activations, the red line represents the group with 25 to 49 activations, the purple line represents the group with 50 to 99 activations, and the blue line represents the group with 100 or more activations

While the number of CFRs roughly remained the same, the number of experienced CFRs increased and the number of less experienced CFRs declined over the study period (Fig. 3). A Spearman’s correlation was conducted to assess the relationship between experience and response intervals and a significant negative correlation was found (*p* < 0.001, Spearman’s rho = -0.0914).

A multiple regression was run to predict CFR response interval from experience, AED, year, time of day, season, Rudkoebing vs. the rest of Langeland. CFR response interval was significantly inverse correlated with experience. Bringing an AED, nighttime and summer were associated with significantly increased response intervals and the town of Rudkoebing significantly reduced response intervals (Table 3). These variables statistically significantly predicted CFR response interval, F (13, 7259) = 17,71, *p* < 0.001, *R*^2^ = 0,031 (Appendix Table 4).

**Table 3:** Results of the regression analysis of 7273 VFR activations, with the variables of community first responders experience, arrival with an AED, the year of the emergency, time of day, season of the year and if it happened in Rudkoebing or not. The variables for the year of 2012, spare time and spring were omitted in the regression analysis

CFR response interval in seconds	Coef	95% Conf. Interval		*P*-Value
Experience of CFR	-0.61	-0.79	-0.43	0.00^*^
Bringing an AED	19.85	4.40	35.31	0.01^*^
Year of 2012	Omitted			
Year of 2013	-33.80	-66.22	-1.38	0.04^*^
Year of 2014	-25.96	-57.97	6.04	0.11
Year of 2015	22.44	-9.98	54.86	0.18
Year of 2016	65.40	33.59	97.20	0.00^*^
Year of 2017	21.47	-13.36	56.30	0.23
Spare time	Omitted			
Work time	3.95	-12.42	20.32	0.64
Night time	104.93	84.68	125.19	0.00^*^
Spring	Omitted			
Summer	25.07	4.61	45.52	0.02^*^
Fall	15.90	-4.95	36.74	0.14
Winter	6.73	-14.76	28.22	0.54
Town of Rudkoebing	-33.75	-49.64	-17.86	0.00^*^
Constant	375.64	344.20	407.08	0.00^*^

^*^ An asterisk represents significant results with a *p*-value of less than 0.05

Official average ambulance response interval on Langeland was 11:18 min and 12.5% of ambulances arrived after more than 20 min, for comparison the official average ambulance response interval in the Region of Southern Denmark was 7:54 min and 1.2% arrived after 20 min [11].

## Discussion

Results from this study of response interval in CFRs show a significant correlation between experience and response intervals. While it seems that there is no substantial difference in median response intervals for CFRs with less than 50 calls accepted and arrived, the most experienced CFRs had median response intervals that were more than one minute shorter when compared to the group with minor experience (Fig. 2). This comparison is strengthened by the good balance of all the group sizes. Increasing experience was negatively correlated to response intervals when analysed according to picking up an AED or time of day through the experience groups. The significant negative correlation between CFR response interval and increasing experience could be replicated when using multiple regression (Table 3). Picking up an AED on the way to the emergency site increased median response intervals by 20–40 s. Increased experience had an impact on median response interval depending on the time of day thus activations at night were reduced by 1:43 min, while working hours was decreased with 1:08 min and spare time with only 46 s when comparing the lowest experienced group with the most experienced. Only the season of summer did impact response intervals significantly, which may be due to increased number of tourists and therefore more traffic and/or because some CFRs are on vacation leaving fewer CFRs on the island to accept the calls. The number of CFR activations is approximately the same (spring 108 pr. month vs. summer 111 pr. month vs fall 105 pr. month vs. winter 101 pr. month). The town of Rudkoebing had a significant shorter CFR response interval compared to the rest of the more rural Langeland. The multiple regression had a low r-squared, indicating that more variables are needed to fully verify the association between experience and response intervals.

In our multiple regression analysis, the town of Rudkoebing was a significant factor in predicting response intervals. Rudkoebing is the most densely populated area and could have interfered with our data since VFRs in Rudkoebing could have had more emergency calls and shorter travel distances. To determine if the negative correlation between experience and response intervals was a result of faster response intervals in Rudkoebing we decided to carry out a multiple regression without the data from the town of Rudkoebing to see if the negative correlation could be replicated in the rural region of Langeland. In the multiple regression without the town of Rudkoebing, the correlation between experience and VFR response intervals is still significant (data shown in Appendix Tables 4, 5 and 6).

More experience could mean that CFRs establish routines to quickly get to the emergency site as they probably are better prepared with established routines and could have an increased local knowledge of AED placements and local addresses. Another possible cause for the decrease in response intervals with increasing numbers of activations could be that the most dedicated CFRs are also the ones with the fastest response intervals. As they possibly are more dedicated, they would probably stay in the project for a longer period meaning that the shorter response intervals found could be a result of the less dedicated CFRs leaving the project before getting experienced. However, we do not believe this is the cause, as a steady decline in unexperienced CFRs over the study period makes it unlikely that a large amount of CFRs left and entered the program (Fig. 3). Also, the total number of CFRs did not change much either while the number of experienced CFRs on Langeland increased over the years which further makes this unlikely (Fig. 3).

Leadership, training, quality improvement and culture of excellence are four important elements deemed essential to support the chain of survival. These four elements are described as the frame of survival and made by the global resuscitation alliance as a tool to define a high-quality EMS system [8]. The frame of survival is also reflected in the Utstein Formula for survival, which states that medical science, educational efficiency and local implementation together equal survival [12]. GPS-based programs like the one initiated on Langeland are a way to provide and improve these four elements from the frame of survival. Strong leadership is shown by implementation of systems to reduce response intervals which multiple countries have done over the last years [13]. The CFRs on Langeland are trained by experienced professionals before they can be activated as part of the system. Leadership and training are fundamentals of the frame of survival that could reduce response interval to increase the survivability of critically ill patients.

No study has explored the correlation between the experience of CFRs and their response intervals but multiple studies have been made with application-based systems [2–7]. In comparison, the median response interval from when the system alerts the CFR to CFR arrival at the emergency site, the Swedish study from Berglund et al. reported 2:22 min for their lay responders to arrive on-site [14] compared to 5:29 min in our study, although we found a median response interval of 4:05 min if only counting the first CFR on-site. For a lay responder to arrive with an AED, Berglund et al. reported 5:17 min in median response time [14] while an AED arrived on-site after 5:45.5 min in our study. A possible explanation for their faster response times could be their higher population density (347 inhabitants/km^2^ in Stockholm County vs 48 inhabitants/km^2^ on Langeland). Our results also support this hypothesis as Rudkoebing which is the biggest town on Langeland has shorter response intervals compared to the rest of Langeland. We believe a higher population density results in shorter response intervals caused by shorter CFR travel distances as more CFRs are available in a higher populated area. Another possible explanation for our longer response intervals is a bigger search radius in our application, as their system used a search radius of 1200 m without AED and 2400 m with AED [14], compared to the Langeland GPS system with a maximum search radius of 5000 m. Thirdly, their system did not activate during nighttime which both our study and another study have shown to increase response intervals [4].

On Langeland in the period of 2012 to 2017 a CFR arrived before the ambulance on the emergency site in 85% of cases [10]. Since studies have highlighted the importance of early CPR [15–17], this study found that the first CFR was on-sight after 4:05 min and thereby describes an effective way to counter the effect of a long EMS response interval. We also found it as a reliable system since a CFR arrived in 98.1% of the calls but this could be extraordinary as a result of the island’s community since Berglund et al. reported 58% [14] and Derkenne et al. 18% [5] of CFRs to arrive at the emergency site. Further studies are needed to enlighten the attendance of CFRs to arrive on-site in GPS based systems, but GPS based systems could be efficient at providing community first responders at emergency sites.

Our data suggest that CFR experience is an important factor in improving response intervals. This could potentially increase survival after a time-critical incident such as an out of hospital cardiac arrest, especially in a rural area with prolonged ambulance response intervals like Langeland. More experienced CFRs would probably also possess better first aid skills and thereby improve the outcome. Continued training and focus on keeping the CFRs active should therefore not be underestimated.

## Limitations

Since the island of Langeland is a small rural part of Denmark, data may not be applicable to other regions or countries. Given no other studies to our knowledge have explored the correlation between experience and response intervals more studies are needed to substantiate our theory. Some CFRs arrived further than 50 m away from the destination resulting in the GPS system not registering them as arrived despite them helping at the location [18]. If a first responder for example, stayed at the cross-section to indicate the direction for the ambulance, they may not register as arrived at the destination. Some data may also have been lost as the system does not register the CFR arrival time if the GPS signal was missing. This could result in some data missing, shown the system as less reliable than in reality and affected CFRs experience and made some CFRs less experienced than they actually were.

## Conclusion

This retrospective study found a significant inverse correlation of CFR experience and response intervals. Using the Langeland GPS system, at least one CFR arrived on-site in 98.1% of all calls and the response interval for the first arriving CFR was 4:05 min. To our knowledge, no other studies have explored the correlation between CFR response intervals and their experience, thus further research is needed to confirm our findings as it could lead to increased survival after a time-critical incident.

## Data Availability

The data that support the findings of this study are available from *Langeland AED Association* but restrictions apply to the availability of these data, which were used under license for the current study, and so are not publicly available. Data are however available from the authors upon reasonable request and with permission of *Langeland AED Association*.

## Appendix

**Table 4:** Multiple regression with all data

Number of obs	=	7,273				
F(13, 7259)	=	17.71				
Prob > F	=	0.0000				
R-squared	=	0.0307				
Adj R-squared	=	0.0290				
Root MSE	=	317.8				
CFR response interval in seconds	Coef	Std. Err	t	P > t	[95% Conf	Interval]
Experience of CFR	-0.612	0.0918	-6.67	0.000	-0.791	-0.432
Bringing an AED	19.851	7.884	2.52	0.012	4.397	35.306
Year of 2012	0	(omitted)				
Year of 2013	-33.798	16.538	-2.04	0.041	-66.219	-1.378
Year of 2014	-25.963	16.326	-1.59	0.112	-57.967	6.041
Year of 2015	22.440	16.540	1.36	0.175	-9.983	54.863
Year of 2016	65.395	16.223	4.03	0.000	33.593	97.198
Year of 2017	21.472	17.767	1.21	0.227	-13.357	56.301
Spare time	0	(omitted)				
Work time	3.950	8.351	0.47	0.636	-12.421	20.320
Night time	104.934	10.331	10.16	0.000	84.682	125.185
Spring	0	(omitted)				
Summer	25.066	10.434	2.40	0.016	4.613	45.518
Fall	15.897	10.635	1.49	0.135	-4.950	36.744
Winter	6.728	10.962	0.61	0.539	-14.761	28.216
Town of Rudkoebing	-33.749	8.107	-4.16	0.000	-49.642	-17.856
Constant	375.640	16.041	23.42	0.000	344.196	407.084

**Table 5:** Multiple regression with data from only the rural region of Langeland without the city of Rudkoebing

Number of obs	=	4,280				
F(12, 4267)	=	16.10				
Prob > F	=	0.0000				
R-squared	=	0.0433				
Adj R-squared	=	0.0406				
Root MSE	=	320.35				
CFR response interval in seconds	Coef	Std. Err	t	P > t	[95% Conf	Interval]
Experience of CFR	-0.272426	0.1350501	-2.02	0.044	-0.5371943	-0.0076577
Bringing an AED	14.75215	10.2348	1.44	0.150	-5.313377	34.81768
Year of 2012	-25.2806	23.0588	-1.10	0.273	-70.48785	19.92665
Year of 2013	-71.69923	20.09696	-3.57	0.000	-111.0997	-32.29874
Year of 2014	-79.55314	18.56365	-4.29	0.000	-115.9476	-43.15873
Year of 2015	-59.63197	18.02671	-3.31	0.001	-94.97369	-24.29025
Year of 2016	69.47226	17.07248	4.07	0.000	36.00131	102.9432
Year of 2017	0	(omitted)				
Spare time	0	(omitted)				
Work time	20.74991	11.04962	1.88	0.060	-0.9130884	42.41292
Night time	117.4228	13.27065	8.85	0.000	91.40539	143.4401
Spring	-5.94705	14.4653	-0.41	0.681	-34.30655	22.41245
Summer	19.81668	13.99651	1.42	0.157	-7.623746	47.25712
Fall	-1.989737	14.51671	-0.14	0.891	-30.45004	26.47056
Winter	0	(omitted)				
Constant	397.5636	20.82995	19.09	0.000	356.726	438.4011

**Table 6:** Multiple regression with data only from the town of Rudkoebing

Number of obs	=	2,993				
F(12, 2980)	=	13.17				
Prob > F	=	0.0000				
R-squared	=	0.0503				
Adj R-squared	=	0.0465				
Root MSE	=	308.06				
CFR response interval in seconds	Coef	Std. Err	t	P > t	[95% Conf	Interval]
Experience of CFR	-0.8917481	0.1248579	-7.14	0.000	-1.136565	-0.6469316
Bringing an AED	25.90219	12.16838	2.13	0.033	2.042912	49.76147
Year of 2012	0	(omitted)				
Year of 2013	-4.271656	32.92418	-0.13	0.897	-68.82809	60.28478
Year of 2014	32.06428	32.0071	1.00	0.317	-30.69398	94.82253
Year of 2015	95.91383	30.10684	3.19	0.001	36.88154	154.9461
Year of 2016	25.37502	28.17623	0.90	0.368	-29.87181	80.62185
Year of 2017	13.88488	29.82281	0.47	0.642	-44.59051	72.36027
Spare time	0	(omitted)				
Work time	-12.1764	12.5416	-0.97	0.332	-36.76746	12.41467
Night time	91.04347	16.23459	5.61	0.000	59.21134	122.8756
Spring	3.678819	16.55592	0.22	0.824	-28.78338	36.14101
Summer	20.22615	16.21573	1.25	0.212	-11.56902	52.02132
Fall	26.11696	16.02235	1.63	0.103	-5.299033	57.53295
Winter	0	(omitted)				
Constant	361.1401	29.42197	12.27	0.000	303.4506	418.8295

## Abbreviations

CFR: Community first responder
GPS: Global positioning system
IQR: Interquartile range
EMS: Emergency medical services
CPR: Cardiopulmonary resuscitation
AED: Automated external defibrillator
